# The ultrastructure of spinal cord perivascular spaces: Implications for the circulation of cerebrospinal fluid

**DOI:** 10.1038/s41598-017-13455-4

**Published:** 2017-10-10

**Authors:** Magdalena A. Lam, Sarah J. Hemley, Elmira Najafi, Nicole G. F. Vella, Lynne E. Bilston, Marcus A. Stoodley

**Affiliations:** 1 Faculty of Medicine and Health Sciences, Macquarie University, New South Wales, NSW 2109 Australia; 2 Macquarie University Microscopy Unit, Faculty of Science and Engineering, Macquarie University, New South Wales, NSW 2109 Australia; 3 Neuroscience Research Australia, Barker Street, Randwick, New South Wales, NSW 2031 Australia

## Abstract

Perivascular spaces play a pivotal role in the exchange between cerebrospinal and interstitial fluids, and in the clearance of waste in the CNS, yet their precise anatomical components are not well described. The aim of this study was to characterise the ultrastructure of perivascular spaces and their role in the transport of fluid, in the spinal cord of healthy rats, using transmission electron microscopy. The distribution of cerebrospinal fluid tracers injected into the subarachnoid space was studied using light, confocal and electron microscopy. Perivascular spaces were found around arterioles and venules, but not capillaries, throughout the spinal cord white and grey matter. They contained fibroblasts and collagen fibres, and were continuous with the extracellular spaces of the surrounding tissue. At 5 min post injection, tracers were seen in the subarachnoid space, the peripheral white matter, the perivascular spaces, basement membranes, extracellular spaces of the surrounding tissue, and surprisingly, in the lumen of blood vessels, suggesting trans-vascular clearance. These findings point out an unrecognised outflow pathway for CNS fluids, with potential implications for volume regulation in health and disease states, but also clinically for the detection of CNS-derived biomarkers in plasma, the immune response and drug pharmacokinetics.

## Introduction

Fluid homeostasis in the central nervous system (CNS) is essential for normal neurological function. The flow dynamics and the composition of cerebrospinal fluid (CSF) have been the subject of numerous studies and reviews. More recently, perivascular spaces have become a topic of intense debate with regards to their role in fluid exchange between the brain or spinal cord parenchymal tissue and the surrounding subarachnoid spaces. A large body of evidence indicates that perivascular spaces represent the major route for fluid inflow from the subarachnoid space into the brain and spinal cord^1–5^, and are also the major pathway for solute clearance^6–8^. The anatomical details of their ultrastructure, however, are not well understood. Early electron microscopic studies described perivascular spaces in hamster^9^ and rat^10^ brain but not in the spinal cord, and the physiological significance of these spaces in the transport of fluid and solutes has not been demonstrated. The flow pathways of cerebrospinal fluid in the spinal cord have been previously investigated using a combination of small molecular weight tracers, light and electron microscopic methods, but with the focus on flow in the central canal rather than the perivascular spaces^4,5,11^.

Conflicting views have been reported in the literature regarding the connection of CSF in the subarachnoid space with fluid in the perivascular spaces^12,13^, and the exchange of CSF with the interstitial fluid is not well documented. The direction and detailed pathways involved in the clearance of fluid and solutes from the CNS parenchyma are also uncertain, with some studies suggesting peri-arterial^6,14^ and others peri-venular^2,8^ outflow. The circulation of the CSF along the central canal of the spinal cord and its penetration into the parenchyma along perivascular spaces have been described as rapid in some studies^3,5,11^, while others reported the bulk flow of fluid, within perivascular spaces of both arteries and veins, as slow and variable in its direction^15^.

The aim of this study was to investigate the ultrastructure of perivascular spaces in the spinal cord with a focus on the continuity of flow pathways, and the relationship of perivascular spaces and the pia/arachnoid cells to the walls of blood vessels and the surrounding tissue. The physiological role of these structures in the transport of CSF and solutes was also studied using a range of CSF tracers.

## Results

### Ultrastructure of perivascular spaces

**Figure 1:**
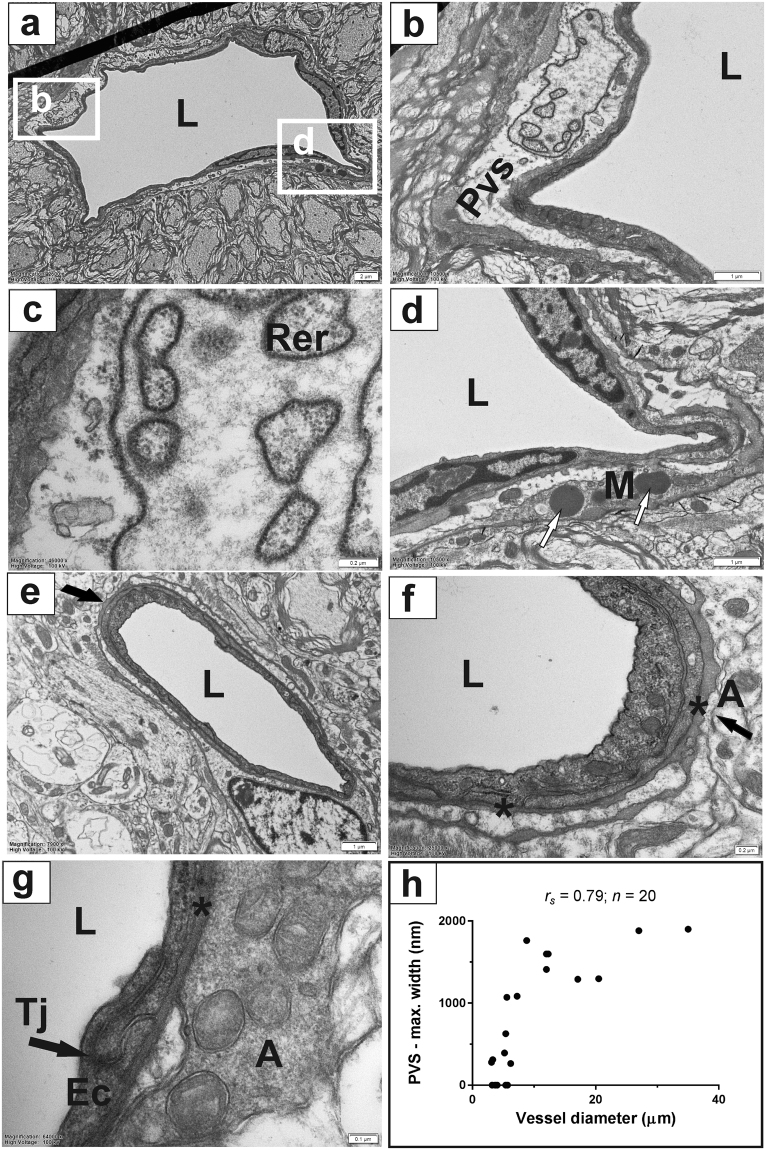
The ultrastructure of spinal cord perivascular spaces. Perivascular spaces accompany arterioles and venules throughout the white and grey matter up to the point where they become capillaries. (**a**) Venule in the peripheral white matter of cervical spinal cord. Perivascular space is present along the entire perimeter of this vessel and is bounded by bordering astrocytes and numerous nerve fibres; (**b**) and (**c**) Higher magnification of an area in A showing the details of pia cell processes with characteristic rough endoplasmic reticulum with associated ribosomes, and collagen fibres in the perivascular space; (**d**) Higher magnification of the inset area in A illustrating a perivascular microglial cell located in between the pia cellular processes and the collagen fibres on the outer border of perivascular spaces; Note the characteristic electron-dense lysosomal bodies (white arrows); (**e**) Low magnification of a vessel in transition between an arteriole and a capillary; arrow is pointing to the portion of the vessel without perivascular spaces; (**f**) Higher magnification of an area in (**e**) showing the portion of the vessel without perivascular spaces, where the endothelial basement membrane (*) and the basement membrane of bordering astrocytes coalesce (arrow). Collagen fibres in the extracellular space (the basement membrane*) can be clearly distinguished in transverse sections; (**g**) Capillary in the central grey matter. The basement membrane is in continuity with the extracellular spaces between bordering astrocytes as well as with intercellular spaces between adjacent endothelial cells up to the occluding tight junction; (**h**) Relationship between the maximum width of perivascular spaces and vessel diameter. A: astrocytes; Ec: endothelial cell; L: lumen; M: microglial cell; Rer: rough endoplasmic reticulum; Tj: tight junction. Scale bars: 2 µm (**a**), 1 µm (**b**,**d**,**e**), 0.2 µm (**c**,**f**), 0.1 µm (**g**).

Our electron microscopic investigations of ultrathin sections of the spinal cord indicated that perivascular spaces were associated with all arterioles and venules, throughout the peripheral white matter and central grey matter of the spinal cord parenchyma. The perivascular compartment was contained between the blood vessel wall and the bordering astrocytes of the spinal cord tissue (Fig. 1a). Cellular processes originating from pia were the main component of the perivascular space with the remaining area filled by tightly packed collagen fibres. The cellular processes of pia were identified by the presence of abundant rough endoplasmic reticulum with multiple ribosomes, characteristic of protein synthesising cells^16^ (Fig. 1b,c). Perivascular microglia were sometimes seen between the pia cellular processes and the outer border of the perivascular spaces and were characterised by oval shaped, electron-dense, lysosomal bodies (Fig. 1d, white arrows).

Larger vessels, arterioles and venules, were entirely surrounded by easily identifiable perivascular spaces. Blood vessels that were intermediate between an arteriole and a capillary, or a capillary and a venule, displayed pia cellular processes surrounding parts of the vessel wall. These structures were adjacent to areas where the basal lamina of endothelial cells and the bordering astrocytes of the surrounding tissue came into direct contact and was the only structure separating the vessel wall from the surrounding parenchyma (Fig. 1e,f, black arrows).

The width of perivascular spaces varied between vessels of different types. To assess the relationship between the width of perivascular spaces and vessel size, the maximum width of perivascular spaces was measured in 20 randomly selected blood vessels and the values were plotted against the vessel diameter. The resulting data (Fig. 1h) indicate that the width of perivascular spaces increases with vessel size, but the maximum width of perivascular spaces observed in the spinal cord did not exceed 2 μm.

Perivascular spaces were not found around capillaries. At this point the basal lamina surrounding a single layer of endothelial cells was in direct contact with the outer plasma membrane of the surrounding CNS tissue, composed primarily of the bordering astrocytes (Fig. 1g). The basal laminae appeared in the electron micrographs as an extracellular space containing extracellular matrix and collagen fibres, and was in continuity with extracellular spaces of the surrounding astrocytes as well as with intercellular spaces between adjacent endothelial cells up to the occluding tight junctions (Fig. 1f,g).

### Ultrastructure of blood vessels and connective tissue in the subarachnoid space

**Figure 2:**
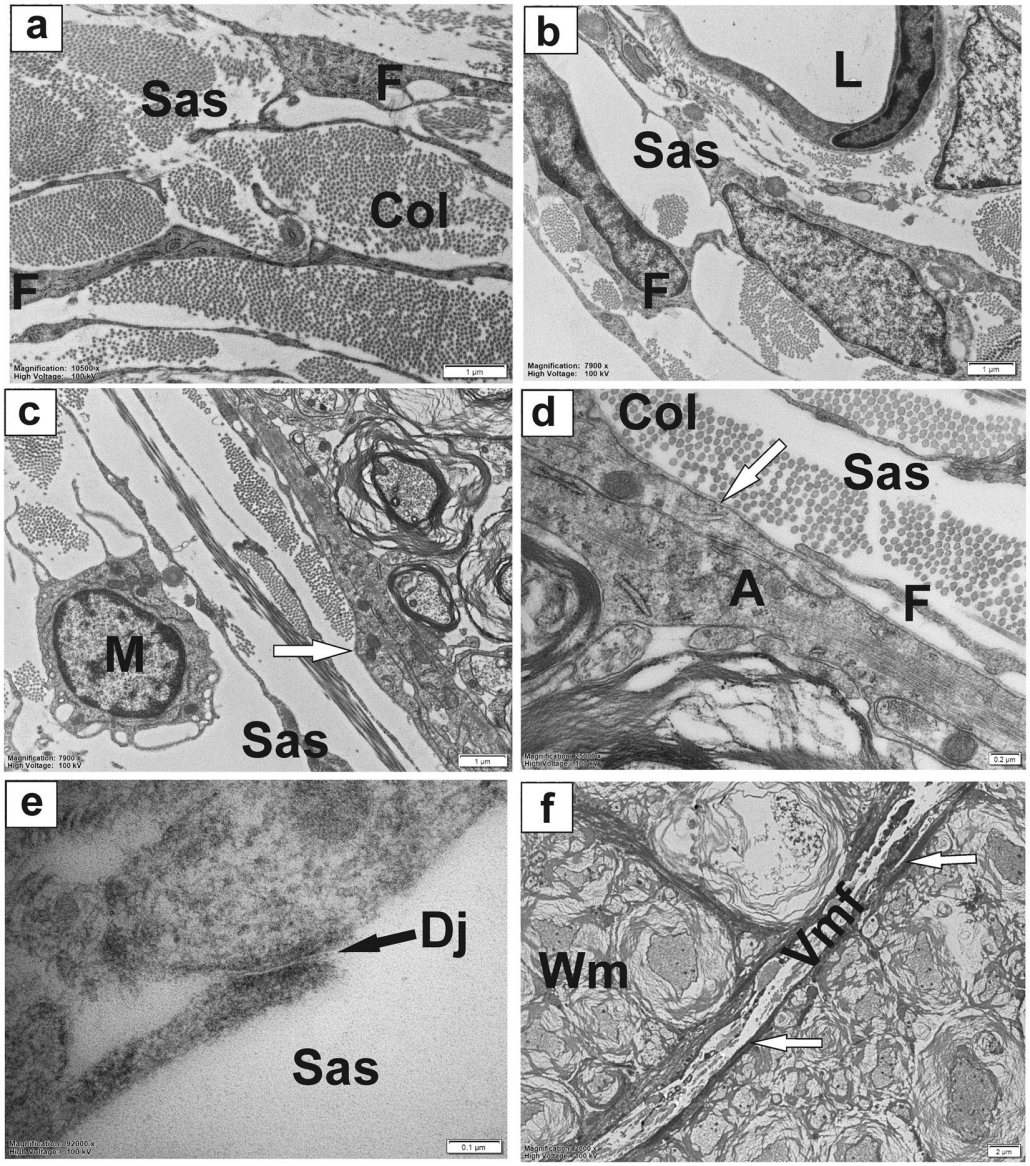
The ultrastructure of spinal subarachnoid space. (**a**) Transverse section from rat spinal cord showing a network of fibroblasts with elongated cellular processes and their characteristic rough endoplasmic reticulum-bound ribosomes, surrounded by bundles of collagen fibres in the subarachnoid space; (**b**) Subarachnoid venule embedded in loosely arranged network of fibroblasts and collagen fibres; (**c**) The interface between the SAS (containing fibrocytes, collagen and a microglial cell) and peripheral white matter of the spinal cord. (**d**) Magnification of an area in (c) illustrating the relationship of pia and extracellular matrix to the bordering astrocytes of the glia limitans. Note discontinuity of the pial cover; (**e**) Desmosome type junction between pia-arachnoid cells in SAS. (**f**) The ventral median fissure of the spinal cord. White arrows: bordering astrocytes (glia limitans). A: astrocyte; Col: collagen; L: blood vessel lumen; F: fibroblast; M: microglial cell; Rer: rough endoplasmic reticulum; Sas: subarachnoid space; Vmf: ventral median fissure; Wm: white matter. Scale bars: 1 µm (**a–c**), 0.2 µm (**d**), 0.1 µm (**e**), 2 µm (**f**).

We next investigated the relationship of intra-parenchymal perivascular spaces with the blood vessels in the spinal subarachnoid space. Transmission electron microscopic analyses of ultra-thin transverse sections demonstrated that the subarachnoid space is primarily occupied by cells of the pia and arachnoid, embedded in a fibrous extracellular matrix (Fig. 2a,b). These cells were identified as active fibroblasts based on a prominent nucleus with peripheral heterochromatin, and well developed rough endoplasmic reticulum and Golgi membranes, indicative of active protein synthesis. In the subarachnoid space, the pia and arachnoid cells formed a network of loosely arranged cells with extensively branched cytoplasmic processes enclosing pools of firmly packed collagen bundles (Fig. 2a). This cellular network, together with the fibrous matrix they produce, seemed to provide support and anchoring for the blood vessels passing through the subarachnoid space (Fig. 2b). We found, however, no evidence of continuous sleeve-like structures accompanying blood vessels in the subarachnoid space. The cellular processes of leptomeningeal cells were close but not in contact with the walls of blood vessels, with frequent gaps or fenestrations (Fig. 2b). The inner most layer of these cells (often referred to as pia mater) was located close to the peripheral white matter (the glia limitans) of the spinal cord. They were, however, separated from the spinal cord glial border by bundles of collagen and a thin layer of electron-dense material associated with the glial plasma membrane, most likely glycocalyx (Fig. 2c,d). Thus, despite anatomical continuity, the true perivascular spaces only exist within the CNS tissue and do not extend into the subarachnoid space beyond the point of entry, or the point of exit, of the peripheral blood vessels.

The cellular processes of pia-arachnoid in the subarachnoid space were sometimes connected by desmosomes or small nexus junctions (Fig. 2e). We found no obvious morphological differences or anatomical separations between pia and arachnoid cells, consistent with earlier views that pia-arachnoid were excavated by the fluid in subarachnoid space rather than forming two distinct limiting membranes^12,17^.

The ventral median fissure appeared as a unique feature of the spinal subarachnoid space, and reached far within the white matter, up to the ventral commissure. It contained cellular processes of pia/arachnoid with associated collagen fibres (Fig. 2f) and was lined by bordering astrocytes (white arrows). It is known as the location of the sulcal arteries and veins that branch off the anterior spinal artery and vein respectively, and split into smaller arterioles and venules as they penetrate the ventral spinal cord parenchyma. These smaller vessels spread radially and are thought to supply most of the ventral grey and white matter. Importantly, they were also accompanied by perivascular spaces.

### Distribution of tracers injected into the subarachnoid space (cisterna magna)

#### *Microscopic analysis of HRP distribution using DAB/H*_2_*O*_2_*reaction*

**Figure 3:**
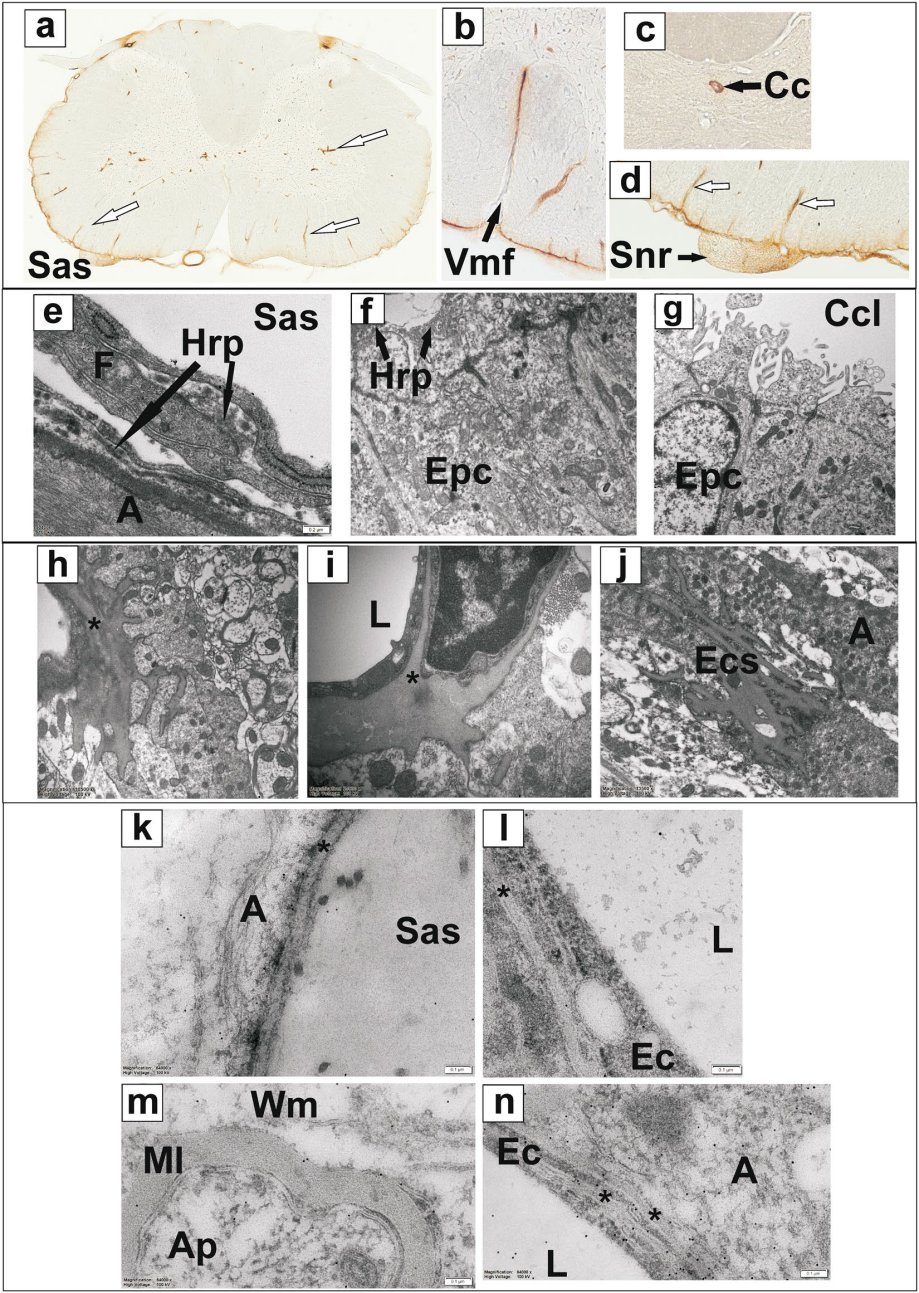
Distribution of cerebrospinal fluid tracer, horseradish peroxidase (HRP). (**a–d**) Visualisation of HRP in cervical spinal cord transverse sections stained with DAB and H_2_O_2_. Intense staining was found in the subarachnoid space and perivascular spaces of penetrating blood vessels (**a**) and (**d**), white arrows, the ventral median fissure (**b**), the central canal (**c**) and spinal nerve rootlets (**d**); (**e–j**) Electron micrographs of ultra-thin sections stained with DAB/H_2_O_2_ and osmium tetroxide. HRP staining is present in the subarachnoid space, associated with leptomeningeal fibroblasts (**e**); and in the central canal lumen as well as in the cytoplasm of ependymal cells of the treated animal (**f**) but is absent in sections from a control animal (**g**). Swelling of the capillary basement membrane* (**h,i**) and extracellular spaces (**j**) in spinal cord sections, due to the oxidising properties of H_2_O_2_ enhanced by the peroxidase activity of HRP; (**k–n)** immuno-gold detection of HRP tracer in ultra-thin sections, showing gold nanoparticles (10 nm) in the subarachnoid space and glia limitans and peripheral white matter (**k**), vascular basement membranes and endothelial cells as well as vascular lumen (l, n) and spinal cord parenchyma (**m**). Ap: axoplasm; Cc: central canal; Ccl: central canal lumen; Epc: ependymal cell; F: fibroblasts; Hrp: horseradish peroxidase; L: blood vessel lumen; Ml: myelin; Sas: subarachnoid space; Snr: spinal nerve rootlets; Vmf: ventral median fissure; Wm: white matter; *basement membrane. Scale bars: 1 µm (**a–c**), 0.2 µm (d), 0.1 µm (**e**), 2 µm (**f**).

The analysis of HRP distribution using DAB/H_2_O_2_ staining in sections from cervical and thoracic spinal cord indicated that 5 min after the end of the injection, tracer was present predominantly in the subarachnoid space. From there, tracer penetrated the parenchyma across the pia and the bordering astrocytes (Fig. 3a). The depth of peripheral staining gradually decreased with increasing distance from the injection site. Intense staining was also observed in the perivascular spaces of penetrating blood vessels extending between the subarachnoid space and central grey matter, as well as in the region of spinal nerve rootlets, the ventral median fissure, and the central canal (Fig. 3a–d). This pattern of staining was consistent with preferential flow along the subarachnoid space, central canal and perivascular spaces. Throughout the spinal cord, the tracer appeared to be spreading from the perivascular spaces into the surrounding parenchyma. The direction of perivascular and trans-parenchymal flow could not be unambiguously determined based on the single time-point investigated.

For more precise localisation of tracers with respect to the vessel wall and perivascular spaces, spinal cord sections stained with DAB/H_2_O_2_ were further processed for electron microscopy as described in methods. The HRP staining was identified in the subarachnoid space (Fig. 3e) and in the central canal lumen (Fig. 3f), but it was not found in sections from a control animals, not injected with HRP (Fig. 3g). Within the spinal cord tissue, the intensity of HRP reaction product staining enhanced with osmium tetroxide was variable, and could not be easily distinguished from the general osmium tetroxide staining of cellular membranes and other lipid-rich structures within the spinal cord tissue. Of interest, however, was the severely swollen appearance of the basement membranes of capillaries and the extracellular spaces in the surrounding tissue (Fig. 3h–j). The swollen membranes were in continuity with the extracellular spaces of the surrounding parenchyma. As the swollen structures were only observed in sections stained with DAB/H_2_O_2_, and were present in both HRP treated and to a lesser extent in control animals, they were attributed to the mildly oxidising nature of H_2_O_2_, which in HRP-treated animals was amplified by the peroxidase activity of the enzyme.

Based on the above difficulties, the distribution of HRP was also studied in two separate animals using immunogold labelling and TEM. In these animals the tracer was identified in the subarachnoid space, and peripheral spinal cord tissue, including bordering astrocytes, and along white matter nerve fibres (Fig. 3k–n). The immunogold staining for HRP was also noted in the endothelium (Fig. 3l,n), in the central canal lumen and in the cytoplasm of ependymal cells, but was not found in control sections where the primary antibody to HRP was omitted in the staining protocol. Overall, the distribution of immunogold was consistent with the distribution of DAB/H_2_O_2_ staining observed using light microscopy, but seemed more uniform across the tissue, without preferential staining of perivascular spaces.

#### CSF flow studies using a protein-fluorophore conjugate

**Figure 4:**
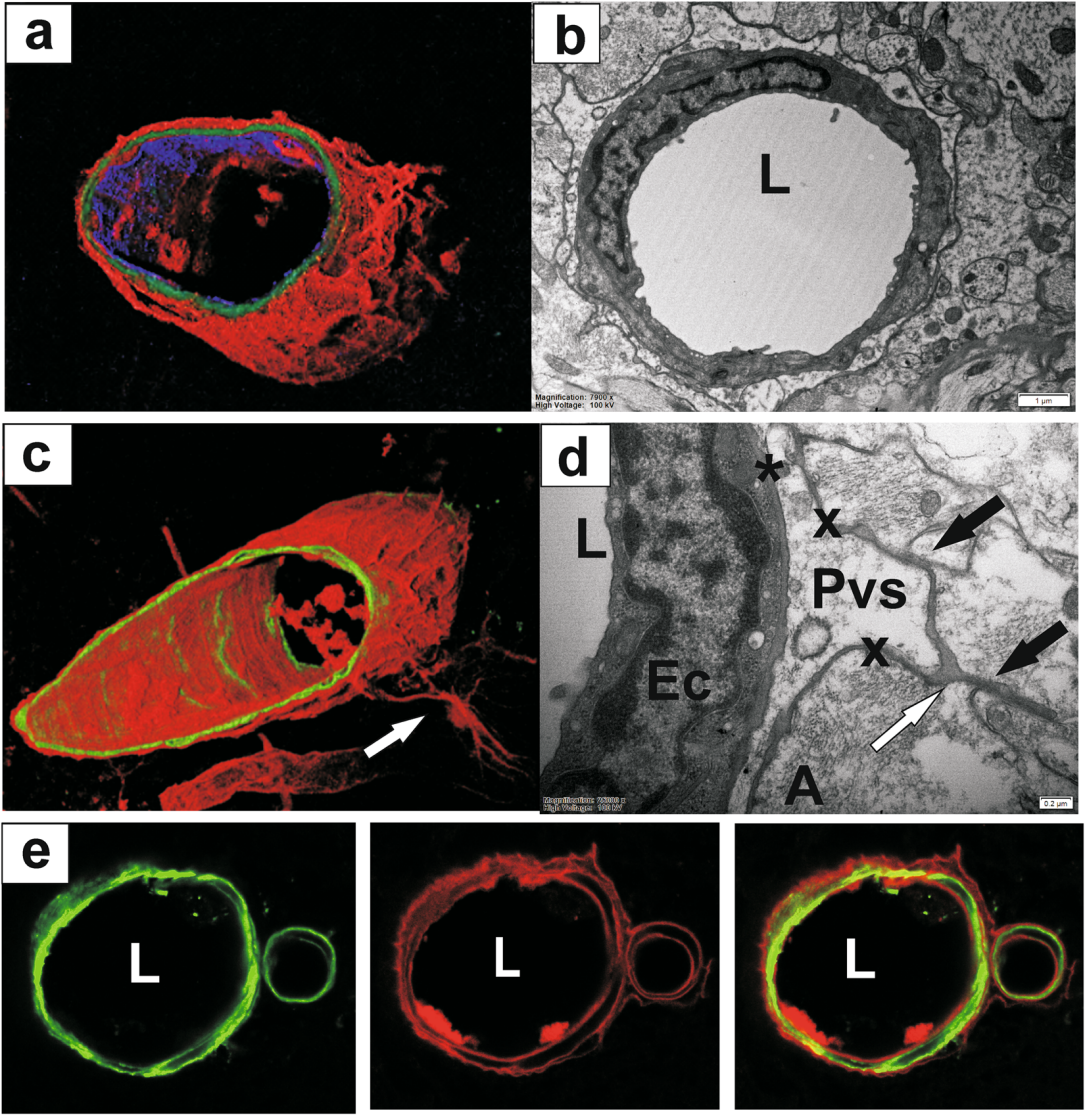
Distribution of cerebrospinal fluid tracer, Ovalbumin-Alexa Fluor 647 (OAF-647). (**a**) and (**c**) Localisation of fluorescent tracer (ovalbumin Alexa Fluor-647; shown in red) in 3D reconstructed images of a vessel in the spinal cord central grey matter; endothelial staining (blue) and smooth muscle actin (green). (**b**) and (**d**) Electron micrographs of similar type vessels in the spinal cord grey matter. Note the distribution of tracer in (**a**) and (**c**) seems to follow the internal and external basement membranes associated with PVS, as well as the extracellular spaces between the bordering astrocytes. Tracer was also found on the luminal side of the smooth muscle cells and even endothelial cells, suggesting clearance across the endothelium into the circulation. (**e**) Higher resolution images of an arteriole illustrating the distribution of tracer (OAF-647 shown in red (*centre*) with respect to the SMA staining (green) (*left*), and the superimposed images (*right*). Ec: endothelial cell; L: blood vessel lumen; Smc: smooth muscle cell. *Basement membrane (internal); x: basement membrane (external).

The location of a fluorescent tracer (OAF-647) with respect to the walls of blood vessels was investigated using confocal microscopy and representative images were matched with electron micrographs from vessels of similar type and size. Near arterioles, the tracer was predominantly seen in the basement membranes surrounding the smooth muscle cells and along the outer border of the perivascular space (Fig. 4a,c). In the electron micrographs, the outer border of perivascular spaces was often found in contact with the glia limitans and the extracellular spaces between bordering astrocytes (Fig. 4b), and it extended into the extracellular spaces of the surrounding tissue (Fig. 4d). The fluorescent tracer was frequently found in these extracellular spaces and connected with tracer present in vascular basement membranes. Surprisingly, tracer was also present in the blood vessel lumen (Fig. 4a,c,e).

#### Distribution of nanoparticle tracers

**Figure 5:**
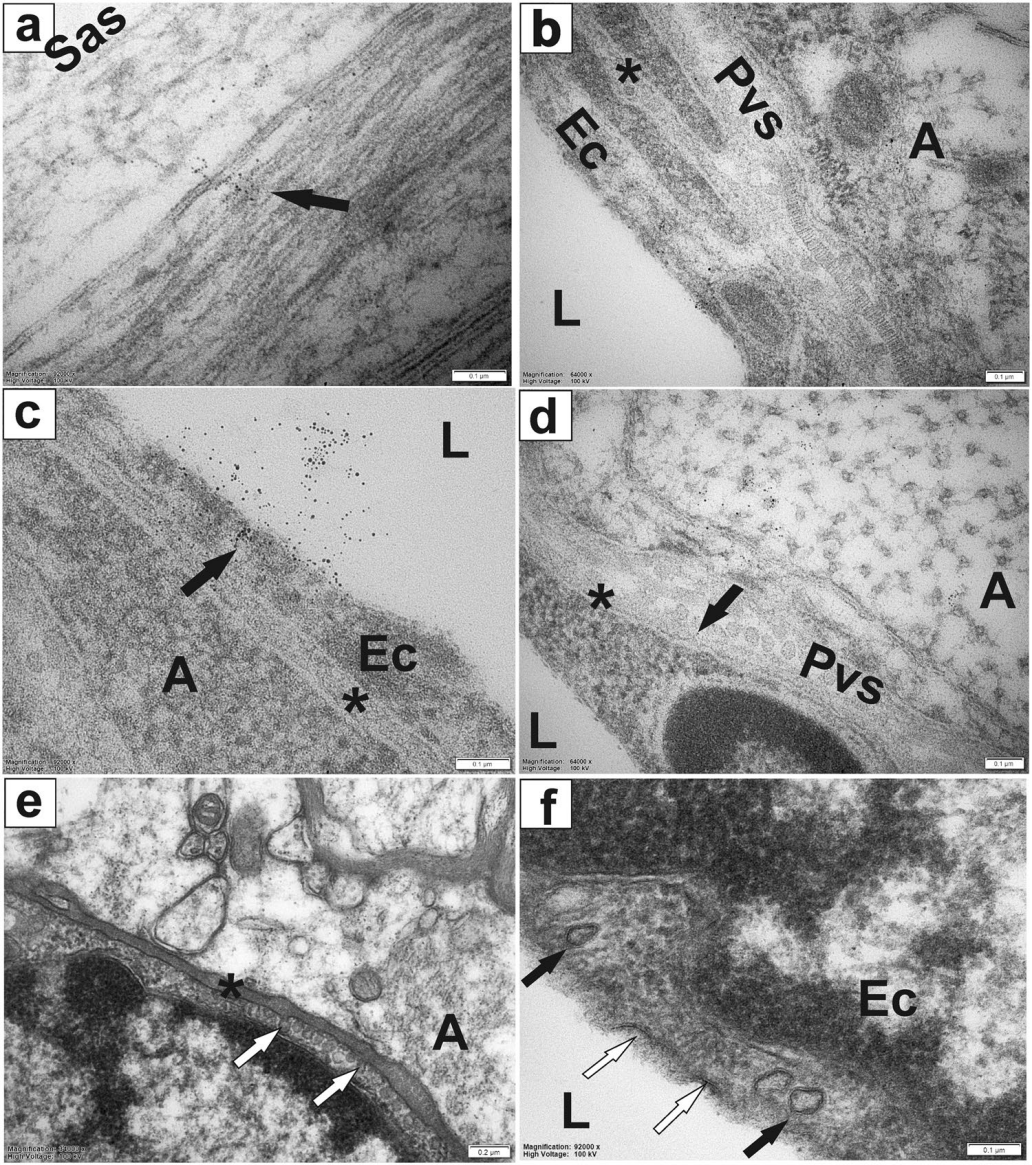
Distribution of cerebrospinal fluid tracer, spherical gold nanoparticles (5 nm). Distribution of spherical gold nanoparticles in the spinal cord 5 min after injection at the cisterna magna. Electron micrographs of ultra-thin, transverse sections from cervical spinal cord showing nanoparticles in the peripheral white matter (**a**), in peri-arterial spaces and vascular wall, endothelial cells, basement membranes and bordering astrocytes (**b**), clearance of tracer across the capillary endothelium into vascular lumen (**c**). Nanoparticles in the bordering astrocyte and perivenular space (**d**). Ultra-thin sections stained with osmium showing vesicles budding off the outer basement membranes of a capillary (e, white arrows) and moving through the endothelium (f, black arrows) and opening into the lumen/fusing with the plasma membrane on the luminal side of endothelial cells (f, white arrows). A: astrocytes; Ec: endothelial cell; L: blood vessel lumen; Pvs: perivascular space; Sas: subarachnoid space; *basement membrane. Scale bars: 0.1 µm (**a**–**d**,**f**), 0.2 µm (**e**).

To further investigate the location of CSF tracers at the ultrastructural level, we used gold nanoparticles as the fluid tracer in combination with transmission electron microscopy. Five minutes after the end of the injection, spherical gold nanoparticles were distributed throughout the cervical and thoracic subarachnoid spaces and spinal cord tissue, with no discernible pattern and no apparent preference for any anatomical structures or pathways. The particles were present in the subarachnoid spaces, peripheral white matter astrocytes, white matter nerve fibres, perivascular spaces associated with both arterioles and venules, basement membranes and endothelial cells (Fig. 5). Particles were also frequently found in the vessel lumen, suggesting clearance of gold particles into the vasculature, most likely via vesicular transport across the endothelium (Fig. 5c). In samples stained with osmium, particles were difficult to discern from the background, but provided additional contrast for visualisation of intracellular vesicles (Fig. 5e,f). While the spherical nanoparticles (5 nm) were distributed uniformly across the spinal cord parenchyma and subarachnoid space, the distribution of nanorods (10 × 20 nm) was restricted to the spinal subarachnoid space. Overall, compared to the distribution of protein tracers primarily in the subarachnoid spaces, the perivascular spaces and the ventral median fissure, the distribution of spherical gold nanoparticles was more uniform across the CNS.

## Discussion

This study investigated the ultrastructure of perivascular spaces in the rat spinal cord as well as their physiological role in the transport of cerebrospinal fluid in the CNS. We confirmed that perivascular spaces are anatomical structures contained between the walls of blood vessels and the surrounding astrocytes in the brain and spinal cord tissue. Furthermore, our observations in the rat spinal cord indicate that these compartments are packed with cellular processes of meningeal cells (the pia and arachnoid), bundles of tightly packed collagen fibres and, less frequently, perivascular microglia. These findings are consistent with earlier studies of perivascular spaces in the brain of cats^12^ and rats^15^. The discontinuity of pia and arachnoid layers allows CSF and solutes to move through the subarachnoid space and enter the perivascular spaces, which are in continuity with subarachnoid and subpial space, consistent with previous reports on similar structures in the brain^12,15,18^. The presence of meningeal cells and fibrous material in the perivascular spaces may indicate that the movement of solutes and fluid occurs mostly via the outer borders of these spaces, specifically the basement membranes. This is supported by the observation that in this and other studies, the distribution of tracers is primarily confined to the outer borders of the perivascular spaces, namely the basement membranes^3,11,19–21^. In the spinal cord parenchyma, perivascular spaces are continuous with the extracellular spaces of the surrounding tissue as well as with the basement membranes of vascular walls. This anatomical continuity provides a low resistance access pathway for subarachnoid fluid and solutes to enter the nervous tissue, and to mix and exchange with interstitial fluid, or to access the vascular basement membranes, consistent with earlier observations of tracer distribution in the brain^3,15^ and spinal cord^11^. Our results are also consistent with earlier reports on the continuity of perivascular spaces with the outer subarachnoid space^9,10,12^ and with the observed rapid penetration of fluid tracers from subarachnoid spaces into the brain or spinal cord tissue via perivascular pathways^2,3,5,22,23^. Furthermore, the continuity of the subarachnoid space with the dorsal and ventral spinal nerve rootlets suggests the existence of an additional outflow pathway for fluid towards the peripheral tissue, along lymphatic or venous systems. This is supported by the observation of intense staining of tracer molecules (HRP or OAF-647) in the ventral and dorsal nerve rootlets, and is consistent with previous reports on the clearance of tracer molecules from the CNS to cervical lymphatics along cranial and spinal nerves^19,24–28^.

An important finding of this study is the possibility of vesicular transport of tracer molecules across the vascular wall (both the smooth muscle and endothelial cells) and clearance to the blood circulation. Similar observations have previously been reported in the literature^11,21,29^, but did not receive much attention from the scientific community. In the spinal cord, endothelial cells of sub-ependymal capillaries were shown to incorporate HRP into pinocytotic vesicles, suggesting trans-vascular clearance, that could be responsible for rapid removal of this tracer from the spinal cord^11^. In the brain, retrograde vesicular transport of HRP through the endothelium, from the basement membrane to the vascular lumen, was also demonstrated, after injection of tracer into the lateral cerebral ventricle^21,29^. Wagner and collegues^21^ proposed that a distinction should be made between fluid and solute transport from the blood to the brain tissue, and the transport in the opposite direction (*i.e*., brain to blood). While there is a well-established requirement for the active regulation of transport of molecules and cells from the peripheral circulation into the CNS (and the free passage of blood borne molecules, including tracers injected *iv*, is limited by the BBB), active transport in the opposite direction may be required for the removal of metabolites and toxic substances, as well as for the regulation of fluid volume. Indeed, previous studies suggest that in the mouse brain, up to 85% of amyloid beta peptide is eliminated by trans-vascular clearance under normal physiological conditions, while a smaller percentage is cleared via other routes, including interstitial fluid bulk flow, followed by transport across the blood brain barrier into the circulation^30–32^. Our observations of trans-vascular CSF tracer clearance suggest that trans-endothelial transport may also be occurring in the spinal cord. Although the mechanism of this transport has not been investigated in detail here, trans-vascular endocytosis with or without the involvement of a non-specific receptor is a likely scenario.

Based on morphological and anatomical differences between the brain and the spinal cord^33^, transport mechanisms and pathways across these two interfaces may differ. Accordingly, it is not yet clear to what extent the findings from this study in the spinal cord will be relevant to the brain. Previous studies in animal models and *in vitro*, suggest that specific differences in the ultrastructure of the BBB and BSCB (blood-spinal cord barrier) may be responsible for the physiological differences between the two barrier systems, including a propensity for certain pathological conditions^33^. The main differences reported so far include the presence of glycogen deposits in the spinal cord but not brain endothelial cells^34^, and increased permeability of spinal cord (compared to brain) to intravenously infused tracers, [3 H]-D-manitol and [14 C]-carboxyl-inulin^35^, and interferons^36^. Based on intrinsic anatomical and functional differences between the brain and spinal cord, regional differences in the BBB and BSCB may exist, but further studies are needed to confirm the preliminary findings.

Recently, it has been proposed that water filtration across the walls of capillaries generates osmotic counter-pressure, essential for the subsequent reabsorption of CNS fluid back into capillaries and venules^37^. Despite existing evidence, the possibility of transport across the vascular wall has been largely discounted. Our observations indicate that vascular endothelial cells in the spinal cord possess both tight junctions and cytoplasmic vesicles, indicative of transport and clearance across cells of the vascular wall. While it is possible that vesicular transport is induced by the presence of exogenous tracer, electron transparent, fluid filled vesicles can also be identified in vascular cells from control animals, suggesting that active transport across endothelial cells occurs in normal physiological conditions and should be considered as a potential outflow pathway from the CNS. The existence of such a pathway would have important implications not only for volume regulation and removal of metabolites but also clinically for the detection of CNS-derived biomarkers in plasma, for the immune response and for drug pharmacokinetics.

Perivascular fluid flow represents a critical component of CSF physiology but many of its aspects remain to be clarified. Recently, the existence of bulk fluid flow through the CNS has been proposed, based on the observations of fast perivascular transport of fluorescent tracers in the mouse cortex^2,8,38^. Termed the glymphatic system, this bulk flow directed from arterial to venous perivascular spaces has been suggested to play an important role in the clearance of metabolites from the CNS parenchyma, with more efficient removal during sleep, and reduced clearance in AQP4 deficient animals^2,39^. This concept has since been questioned by other investigators^40,41^ who have shown that arterial pulsations are unlikely to generate substantial bulk fluid flow, but may generate local fluid motion that leads to fast perivascular solute transport by dispersion (i.e., a combination of mixing and diffusion)^40^. Such a model is in agreement with observations of reduced perivascular tracer movement after aortic ligation^4,42^ or occlusion^3^, both of which are expected to reduce arterial pulsations and thereby limit dispersion. The distribution of tracers around vessels observed in this study supports the dispersion model, and further suggests that the main anatomical structures involved in the transport of fluid and solutes across the spinal cord tissue are the vascular basement membranes. The volume capacity of these structures depends on the size of the blood vessel, with arterioles showing the greatest capacity due to multiple layers of basement membrane (endothelial, and one or two additional layers surrounding the smooth muscle cells), followed by venules (one to two layers) and capillaries (only one layer). The vascular basement membranes are in continuity with the extracellular spaces of the surrounding tissue providing access for fluid and small solutes into the adjacent parenchyma, including the white matter and central grey matter of the spinal cord, all the way to the central canal. Compared to the rapid flow from the subarachnoid space into the perivascular spaces, the subsequent movement of solutes from the basement membrane into the narrow gaps between astrocytic processes, (typically 10–20 nm in width) is expected to be much slower, and largely dependent on the molecular size and charge. This is supported by the observation that 5 nm spherical gold particles, and protein tracers (HRP and OAF-647, 40–45 kD) penetrated the spinal cord from subarachnoid spaces to central canal, whereas 10 × 20 nm gold nanorods remained in the subarachnoid spaces after cisterna magna injection, consistent with earlier reports^15^. A recent study of CSF flow pathways in mice demonstrated that in the brain, vascular basement membranes are the major routes for fluid flow into and out of the brain and that movement of solutes is limited by molecular size^20^. Based on the distribution of fluorescent amyloid beta and gold nanoparticles (15 nm), the authors proposed that separate pathways exist for the entry and drainage of fluid with entry via pial-glial membrane and drainage initially via capillary basement membrane followed by smooth muscle cell basement membrane of arterioles^20^. However, these differential pathways observed in the brain were likely due to size limitation (restricted movement of 15 nm nanoparticles) rather than real differences in fluid pathways. Our data do not support such separation of inflow and outflow pathways. The distribution of protein tracers (HRP and OAF-647) was consistent with preferential flow through subarachnoid space, and entry along perivascular spaces of both arterioles and venules, and via the central canal. The distribution of 5 nm nanoparticles was in general more uniform throughout the spinal cord tissue with no apparent preference for perivascular location, although the movement of spherical particles is possibly faster compared to bulky proteins. Overall these results support the notion that the movement of CSF tracers is dictated by their molecular size and charge.

Currently, peri-arterial spaces are an established route of fluid entry, but there is debate regarding the routes of fluid clearance from the CNS tissue with both peri-arterial^6,14^ and peri-venular^2,8^ outflow suggested. Existing views are largely based on the spatial distribution and temporal variation of tracer molecules of different molecular size, after injection into the tissue or fluid filled spaces in brain or spinal cord. Interpretation of these types of experiments requires caution as artefacts can be introduced due to changes in pressure, volume or even the nature of the exogenous tracer molecule. The choice of the time point for tracer imaging is crucial. In the spinal cord, the 5 min time point is suitable for studies of inflow pathways, but a 30 min time point is difficult to interpret because both inflow and outflow processes are likely to be captured and cannot be differentiated. Longer time-points (1–3 h) may seem appropriate for the study of outflow routes, although in our experience most of the tracer is cleared from the tissue within the first hour; any tracer molecules remaining may indicate retention due to interaction of tracer with cell membranes or cellular uptake, mainly by microglial cells^21^. Our electron microscopic investigations of the spinal cord tissue indicated that both peri-arterial and peri-venular spaces are in continuity with the subarachnoid space. Hence, tracers injected in the subarachnoid space (*e.g*. the cisterna magna) should have access to both arterial and venular perivascular spaces, although transport is expected to be faster in peri-arterial spaces due to greater width and the mixing effect of arterial pulsations^1,40^. In our TEM investigations of rat spinal cord, we found no evidence of valve structures or any other anatomical features that would allow flow in only one direction and tracers injected into subarachnoid space (cisterna magna) were found to penetrate both peri-arterial and peri-venular spaces. Therefore, CSF flow through perivascular spaces and basement membranes should be possible in both directions, consistent with the “to- and fro- pulsatile movement” observed *in vivo* ^43^.

Although tracers are used extensively to study the dynamics of CSF flow, the use of exogenous molecules can result in artefacts that don’t reflect physiological processes. Cellular uptake has been described for various exogenous molecules, in particular nanomaterials^44,45^, and contributes to the removal of tracers^21^ from the CSF. The appearance of ‘labyrinth structures’^11^ in ultra-thin sections stained for peroxidase (HRP) is due to swelling of the basement membrane and may be explained by oxidative damage induced by DAB/H_2_O_2_ staining. This is consistent with earlier reports suggesting that compared to cells, the extracellular components are more susceptible to oxidative damage due to lower levels of antioxidant defences and repair systems^46,47^. The intravascular location of tracers could possibly be an artefact generated during the processing of tissue samples, although at this stage, it is not clear which process could lead to this result. It is not likely to be due to perfusion of tissue with the fixative, paraformaldehyde, which is introduced into the ascending aorta, and could only have an opposite effect. The dragging of tracer during sectioning is also unlikely. In the case of gold nanoparticles imaged by electron microscopy, fixed samples are embedded in acrylic resin, polymerised, and the hardened resin blocks are sectioned on an ultramicrotome. The size of the tracers (5–10 nm) relative to the thickness of the section (approx. 70–80 nm) means that statistically, most particles in the section would have resin on both sides of the tracer, preventing them from popping out during sectioning. Also, there was no evidence of poor infiltration (or holes in the sections, which would appear paler), that may allow section constituents to come loose and escape during sectioning. In the case of fluorescent tracer (Ovalbumin-Alexa Fluor conjugate; 45 kDa; approx. 5 nm) imaged by confocal microscopy, fixed samples are embedded, frozen and sectioned (40 μm) on a cryostat. If dragging was occurring, the entire image would appear fuzzy, but this is not the case. A potential limitation of this study is that the diameter of a blood vessel and the size of the perivascular spaces of the rat spinal cord, measured in our electron micrographs, could be affected by sample processing, and is therefore an approximation of the true size. It is nevertheless more accurate than a measure of these spaces using a light microscopic approach. The observation that the size of these spaces (subject to processing) did not exceed 2 µm, remains an interesting finding and can be compared, in the future studies, to the size of perivascular space in processed tissue samples from models of disease. Furthermore, the diameter of a blood vessel and the width of perivascular space would be affected equally by sample processing, therefore their ratio should remain constant.

While our data suggest trans-vascular tracer clearance, based on snap-shot images, the trans-vascular flow, as a dynamic process, would be ideally studied in real-time, *in vivo*, and is one of our future aims. However, the limiting factor of most currently available *in vivo* imaging techniques, including MRI, PET or SPECT, is the relatively low resolution. It is therefore unlikely that an *in vivo* evidence of trans-vascular flow will become available in the near future.

## Conclusion

This study adds to the current understanding of the ultrastructure of perivascular spaces and fluid transport pathways in the central nervous system, showing that perivascular spaces are expansions of the outermost vascular basement membrane, and contain both cellular and fibrous material. The continuity of perivascular spaces with the basement membranes and extracellular spaces of the surrounding tissue facilitates fluid flow and clearance. The finding of vesicular transport across the wall of blood vessels has to be further validated, but could have important implications for fluid outflow and the clearance of macromolecules. The spatial distribution of tracers supports the vital role of perivascular spaces and vascular basement membranes in the transport and removal of fluid, solutes and cells (*e.g*. microglia) through the CNS. Whether and how the ultrastructure of perivascular spaces is altered in CNS diseases that involve fluid imbalance (such as hydrocephalus, oedema and syringomyelia) is unknown, and needs to be investigated.

## Methods

### Transmission electron microscopy

All experimental methods involving animals were performed in accordance with the Australian code for the care and use of animals for scientific purposes, and were approved by the Macquarie University Animal Ethics Committee (ARA 2013/047). The ultrastructure of perivascular spaces in the spinal cord was investigated in five Sprague Dawley rats (250–300 g) using transmission electron microscopy of transverse ultra-thin sections from cervical and thoracic spinal segments. Animals were placed under general anaesthesia using isoflurane (5% for induction, 2% for maintenance). Transcardiac perfusion with 200 mL 0.1 M PBS containing 5000U of heparin was followed by a mixture of 3% paraformaldehyde and 2.5% glutaraldehyde in 0.1 M sodium phosphate buffer, pH 7.2. The spinal cord and brain were removed and postfixed at room temperature for 1 h. Transverse sections (1 mm) from each spinal segment (C2-T4) were incubated in fresh fixative overnight at 4 °C. Samples were postfixed in osmium tetroxide (1%, 1 h) and stained *en bloc* with uranyl acetate (2%; 30 min), followed by dehydration in graded ethanol solutions and infiltration with LR white resin (ProSciTech). Polymerised resin blocks were sectioned on an ultramicrotome (Leica, UC7). Semi-thin sections (750 nM) were stained with a mixture of 1% methylene blue, 0.6% sodium bicarbonate and 40% glycerol, and were used for orientation. Ultra-thin sections (70–80 nm) were dried on coated copper grids, stained with uranyl acetate (7%; 8 min) and Reynolds lead citrate^48^ (3 min) and imaged using a Philips CM10 TEM equipped with an Olympus SIS Megaview G2 digital camera.

### Cisterna magna injection of tracers

Cerebrospinal fluid flow from the subarachnoid space into the spinal cord central grey matter was studied in 10 Sprague Dawley rats using small protein tracers, (horseradish peroxidase [HRP-Type VI A, Sigma Aldrich, 10% solution] and ovalbumin-Alexa Fluor 647 conjugate [OAF; Life Technologies; 20 mg/mL]) or gold nanoparticles (5 nm spheres; 1 mg/mL, or gold nanorods 10 × 20 nm; NanoPartz, 35 µg/mL). Tracers were suspended in sterile saline (0.9% NaCl) and injected in the cisterna magna using a 10 µL Hamilton syringe with a 30-gauge needle, held in a stereotactic frame, and advanced through the exposed atlanto-occipital membrane. A micropump (Ultramicropump III with Micro4 controller, WPI) was used to infuse 10 µL of HRP (10% solution) or gold nanoparticles (1 mg/mL) at a rate of 33 nL/s. The needle was left in place for 3 min after the injection to prevent tracer or CSF loss. Five minutes after the end of the injection, rats were perfused and spinal cord tissue processed as above. To allow for fixation of gold within the spinal cord tissue during aldehyde perfusion, COOH-capped spherical gold nanoparticles were conjugated to lysine (*L*-lysine, Sigma Aldrich) at a molar ratio of 1:1, using the reaction with *N*-hydroxysulfosuccinimide (Sulfo-NHS) enhanced by 1-ethyl-3-[3-dimethylaminopropyl] carbodiimide chloride (EDC). The heavy metal stains, osmium tetroxide and uranyl acetate, typically used in preparation of tissue samples for electron microscopy, can form a fine precipitate and were therefore not used during sample preparation, to ensure particles were easily identified and not confused with precipitating stains. For visualisation of nanoparticles, ultra-thin sections were imaged without or with minimal uranyl acetate and lead citrate staining (8 and 3 min respectively). For visualisation of HRP, tissue was embedded in 1.5% agarose and 50 µM sections were cut on a vibratome. After reaction with diaminobenzidine (DAB, 0.1%) and hydrogen peroxide (H_2_O_2_, 0.02%) in Tris-PBS, pH 7.4 for 15 min, sections were washed and mounted on glass slides with glycerol, cover-slipped and imaged using a Zeiss Axio Imager in brightfield mode. Some sections were further processed for electron microscopy as described above.

### Confocal microscopy

For visualisation of ovalbumin-Alexa Fluor 647 sections were imaged on a confocal laser scanning microscope (LSM880, Zeiss). To determine the location of tracer with regards to the walls of blood vessels, sections were incubated with Rat Endothelial Cell Antibody (1:100; RECA-1; AB9774 Abcam, Cambridge, United Kingdom) followed by a secondary antibody (1:400; anti-mouse IgG Alexa Fluor 488; Molecular Probes, Life Technologies, New York, USA) and with actin *α*-smooth muscle antibody (1:400; SMA-Cy3; Sigma-Aldrich, St. Louis, Montana). Images were acquired in a *z*-stack scan mode, with interval of 0.5 μm, as a 4 line average, pixel dwell 0.76 μs, using a plan-apochromat 40x/1.3 oil DIC UV-IR M27 objective. Image dimensions were *x*: 1024, *y*: 1024, *z*: 36 μm; 8-bit.

### Image processing

3D projections were generated from raw data, using the transparency rendering mode in Carl Zeiss Zen 2012 software (black edition, version 8).

### Immunogold detection of horseradish peroxidase

Immunogold labelling of HRP was carried out on ultra-thin (70 - 80 nm) transverse spinal cord sections using a standard protocol with modifications^49^. Sections were dried on coated nickel grids, incubated with a primary antibody to HRP (1:25; mouse monoclonal anti-HRP, Abcam) followed by a secondary antibody conjugated to 10 nm gold particles (1:250; BBI goat anti-mouse IgG; H&L; EM grade; 10 nm; ProSciTech). Negative controls included sections where the primary antibody was omitted, as well as spinal cord sections from an animal that did not receive HRP injection.

### Data availability

All data generated or analysed during this study are available from the corresponding author on reasonable request.
